# Multiple Reassortants of H5N8 Clade 2.3.4.4b Highly Pathogenic Avian Influenza Viruses Detected in South Korea during the Winter of 2020–2021

**DOI:** 10.3390/v13030490

**Published:** 2021-03-16

**Authors:** Yoon-Gi Baek, Yu-Na Lee, Dong-Hun Lee, Jae-in Shin, Ji-Ho Lee, David H. Chung, Eun-Kyoung Lee, Gyeong-Beom Heo, Mingeun Sagong, Soo-Jeong Kye, Kwang-Nyeong Lee, Myoung-Heon Lee, Youn-Jeong Lee

**Affiliations:** 1 Avian Influenza Research & Diagnostic Division, Animal and Plant Quarantine Agency, 177 Hyeoksin 8-ro, Gimcheon-si 39660, Gyeongsangbuk-do, Korea; bygttaggg@gmail.com (Y.-G.B.); ynlee27@korea.kr (Y.-N.L.); SJI6951@naver.com (J.-i.S.); leejiho5447@naver.com (J.-H.L.); ensenble@korea.kr (E.-K.L.); imheo@korea.kr (G.-B.H.); sagongmg@korea.kr (M.S.); cessna70@korea.kr (S.-J.K.); leekwn@korea.kr (K.-N.L.); vetlee@korea.kr (M.-H.L.); 2 Department of Pathobiology and Veterinary Science, The University of Connecticut, 61 North Eagleville Road, Unit-3089, Storrs, CT 06269, USA; dong-hun.lee@uconn.edu (D.-H.L.); hyunjung.chung@uconn.edu (D.H.C.)

**Keywords:** highly pathogenic avian influenza virus, reassortant, transmission, H5N8, wild bird, domestic duck

## Abstract

During October 2020–January 2021, we isolated a total of 67 highly pathogenic avian influenza (HPAI) H5N8 viruses from wild birds and outbreaks in poultry in South Korea. We sequenced the isolates and performed phylogenetic analysis of complete genome sequences to determine the origin, evolution, and spread patterns of these viruses. Phylogenetic analysis of the hemagglutinin (HA) gene showed that all the isolates belong to H5 clade 2.3.4.4 subgroup B (2.3.4.4b) and form two distinct genetic clusters, G1 and G2. The cluster G1 was closely related to the 2.3.4.4b H5N8 HPAI viruses detected in Europe in early 2020, while the cluster G2 had a close genetic relationship with the 2.3.4.4b H5N8 viruses that circulated in Europe in late 2020. A total of seven distinct genotypes were identified, including five novel reassortants carrying internal genes of low pathogenic avian influenza viruses. Our Bayesian discrete trait phylodynamic analysis between host types suggests that the viruses initially disseminated from migratory waterfowl to domestic duck farms in South Korea. Subsequently, domestic duck farms most likely contributed to the transmission of HPAI viruses to chicken and minor poultry farms, highlighting the need for enhanced, high levels of biosecurity measures at domestic duck farms to effectively prevent the introduction and spread of HPAI.

## 1. Introduction

Since 1996, the Asian-origin H5Nx A/goose/Guangdong/1/1996 (Gs/GD) lineage of highly pathogenic avian influenza (HPAI) viruses has caused outbreaks in poultry and wild birds across four continents [1]. The Gs/GD-lineage H5Nx HPAI viruses have evolved into 10 genetically distinct virus clades (0–9) [2]. During 2013–2014, H5N8 clade 2.3.4.4b HPAI virus was first detected in the domestic duck in eastern China and subsequently reported in South Korea [3,4]. In mid-2016, H5N8 2.3.4.4b HPAI viruses carrying internal genes of Eurasian low pathogenic avian influenza (LPAI) were identified in wild birds at Qinghai Lake, China, and Uvs-Nurr Lake at the Russia–Mongolia border [5,6]. During 2016–2017, the clade 2.3.4.4b HPAI reassortant caused massive outbreaks in wild birds and poultry, and considerable economic damage to many countries [7,8,9,10,11,12]. Since 2018, outbreaks of the H5N8 clade 2.3.4.4b HPAI virus have been reported in Europe, Asia, and Africa [13,14,15,16].

In December 2019, a novel reassortant clade H5N8 2.3.4.4b HPAI virus carrying six gene segments derived from HPAI viruses caused outbreaks in Africa, and two gene segments from Eurasian LPAI viruses were reported in Europe [17]. Subsequently, several outbreaks in poultry and wild birds caused by this reassortant HPAI virus were reported in multiple European countries, including Poland, the Czech Republic, Hungary, and Germany, up to April 2020 [18]. In May 2020, the Republic of Iraq reported a novel H5N8 clade 2.3.4.4b HPAI virus in poultry that is genetically close to the H5N8 2.3.4.4b HPAI virus that circulated in Eurasia during 2017–2018 [19]. The genome constellation of the virus detected in Iraq was distinct from that of the reassortant viruses detected in Europe during early 2020. Furthermore, reassortant H5N1 and H5N5 viruses related to the H5N8 virus reported in Iraq were detected in the Netherlands and Germany [19].

In South Korea, HPAI virus had not been detected since April 2018. In October 2020, H5N8 clade 2.3.4.4b HPAI viruses genetically close to the viruses detected in Europe during early 2020 were detected in wild birds in South Korea [20]. The H5N8 viruses have also been identified in poultry farms since the last week of November 2020. During October 2020–January 2021, we sequenced a total of 67 HPAI H5N8 viruses from wild birds and poultry in South Korea and phylogenetically analyzed complete genome sequences to determine the origin, evolution, and spread patterns of these viruses.

## 2. Materials and Methods

In this study, a total of 13,033 samples from wild bird habitats and 102,812 samples from poultry farms were collected through active surveillance in South Korea from July 2020 to January 2021. In major migratory habitats, oropharyngeal and cloacal swabs were collected from captured birds. Carcasses of birds and fecal samples were also collected. In poultry farms, fecal samples, carcasses, and oropharyngeal and cloacal swabs were also collected. We sequenced 67 HPAI virus isolates (50 poultry and 17 wild birds) identified from South Korea during the winter of 2020–2021. Viral RNA was extracted from samples using the Patho Gene-spin DNA/RNA Extraction Kit (iNtRON Biotechnology, Sungnam, Korea). All 8 segments of isolates were amplified by RT-PCR [21]. The PCR products were purified with AMPure XP magnetic beads (Beckman Coulter, Brea, CA, USA). Multiplexed paired-end sequencing libraries were generated using the Nextera™ DNA Flex Library Prep Kit (Illumina, San Diego, CA, USA) according to the manufacturer’s instructions. Complete genome sequencing was conducted using the Illumina MiSeq next-generation sequencing platform (Illumina, San Diego, CA, USA) according to the manufacturer’s instructions. Genome sequences were directly assembled using the CLC Genomics Workbench software. The sequences of the viruses isolated in this study were deposited in the GISAID database (http://platform.gisaid.org (accessed on 17 February 2021)); the accession number of each virus is listed in Supplementary Table S1 (EPI_ISL_985179-EPI_ISL_985211, EPI_ISL_1009679-EPI_ISL_1009709, and EPI_ISL_631824). A DNA barcoding system utilizing mitochondrial DNA from the fecal specimens was employed to determine the host species of the avian influenza virus (AIV), as previously described [22].

The reference genome sequences used in this study for the phylogenetic analysis were obtained from GenBank (www.ncbi.nlm.nih.gov/genomes/FLU (accessed on 17 February 2021)) and the GISAID EpiFlu database (http://www.gisaid.org (accessed on 17 February 2021)) (Supplementary Table S2). All the available sequence of AIV information was retrieved in December 2020 to generate the maximum-likelihood (ML) phylogenetic trees. For the HA gene, all the available sequences of clade 2.3.4.4b H5 HPAI viruses were collected from the database on January 2021. Among them, the reference sequences of clade 2.3.4.4b H5 HPAI were selected after considering the geographical locations and collection dates. Complete coding sequences of each gene segment were used for comparative phylogenetic analyses. Multiple sequence alignments (PB2: 2280 bp, PB1: 2274 bp, PA: 2151 bp, HA: 1704 bp, NP: 1497 bp, NA: 1413 bp, M: 982 bp, and NS: 838 bp) were prepared using MAFFT [23].

To estimate the time to most recent common ancestor (TMRCA), maximum clade credibility (MCC) trees for the hemagglutinin (HA) gene were constructed using BEAST, version 1.10.4 [24]. We applied an uncorrelated lognormal distribution relaxed clock method, the HKY nucleotide substitution model, and the GMRF Bayesian Skyride coalescent prior [25,26]. We initially reconstructed the phylogenetic tree using all of the clade 2.3.4.4b viruses available in GenBank and GISAID and selected representative sequences for our dataset. Our final dataset for estimating the time of emergence and spread of H5N8 clade 2.3.4.4b viruses in South Korea contained 116 sequences. Three posterior trees having effective sample sizes >200 in Tracer 1.7.1 (http://tree.bio.ed.ac.uk/software/tracer/ (accessed on 17 February 2021)) after 50 million runs were combined using LogCombiner v1.10.4 (https://www.beast2.org/programs/ (accessed on 17 February 2021)). Then, 10% of each run was removed as a burn-in, and MCC trees were generated using TreeAnnotator v1.8.1 (http://beast.bio.ed.ac.uk/TreeAnnotator/ (accessed on 17 February 2021)). The tree was visualized using FigTree 1.4.4 (http://tree.bio.ed.ac.uk/software/figtree/ (accessed on 17 February 2021)). Furthermore, we characterized the contribution of wild and domestic birds to the transmission dynamics of viruses in South Korea. A transition of viruses between host types was estimated using a discrete ancestral state reconstruction method and asymmetric host transitions. We included all the H5N8 HPAI viruses identified in South Korea during October 2020–January 2021 (*n* = 67) and closely related HPAI viruses identified in Europe during 2020 (*n* = 7). The final dataset consisted of 74 taxa, which were coded into four host types: wild birds (*n* = 24), domestic ducks (*n* = 26), chickens (*n* = 19), and minor poultry (quail, geese and white peacock; *n* = 5). We applied a Bayesian stochastic search variable selection to identify the best supported host transitions using a Bayes factor (BF) test. The BF and posterior probability calculated using SpreaD3 v0.9.6. (https://rega.kuleuven.be/cev/ecv/software/SpreaD3 (accessed on 17 February 2021)) were considered significant when >4 and 0.5, respectively. We also calculated the number of transitions between host types (Markov jump) and the times between host type changes (Markov reward) in poultry [27].

To describe the genetic diversity of the H5N8 viruses detected in South Korea during 2020–2021, ML phylogenetic trees for all the genes were generated with RAxML version 8.2.12 using a gamma distribution and a general time-reversible model with all of the AIV sequences available from the GISAID and GenBank databases since 2000 [28]. The genotype was analyzed according to the tree topology and a nucleotide sequence identity of >97%, regarded as significant when the bootstrap support value >80, as described in a previous study [29].

## 3. Results and Discussion

**Figure 1:**
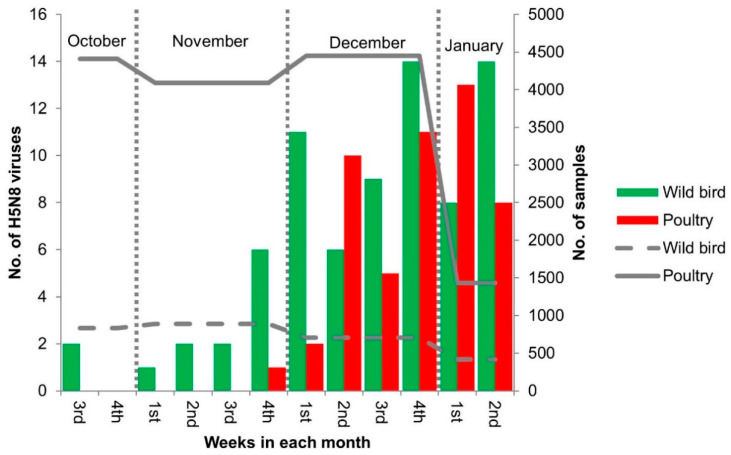
H5N8 highly pathogenic avian influenza viruses isolated from wild birds and poultry in South Korea during the winter of 2020–2021. Bars represent the numbers of H5N8 virus isolates in wild bird and poultry in South Korea according to time from October 2020 to January 2021 (green, wild bird; red, poultry). Lines represent the numbers of sampled wild birds and poultry (dotted line, wild bird; solid line, poultry).

During October 2020–January 2021, a total of 75 HPAI viruses were isolated from wild birds (23 fecal droppings, 37 carcasses, and 15 swab samples of captured birds) in South Korea (Figure 1), including 17 isolates by the Animal and Plant Quarantine Agency (APQA) and 58 isolates by the National Institute of Environmental Research. In addition, since first being identified in a broiler duck farm during active surveillance on November 26, 2020, a total of 50 H5N8 HPAI viruses have been isolated from poultry farms by the APQA as of 9 January 2021, including during outbreaks from 26 duck farms, 19 chicken farms, and 5 minor poultry farms. The detection rates for HPAI viruses isolated in poultry and wild bird habitats were 0.04% and 0.1%, respectively. In the present study, the complete genome sequencing and comparative phylogenetic analysis of 67 H5N8 HPAI viruses isolated from poultry and wild birds in South Korea during the winter of 2020–2021 were conducted to better elucidate both the source and transmission dynamics within South Korea. All the isolates sequenced in this study possessed multibasic amino acid sequences (PLREKRRKR*GLF (*n* = 65) and PLIEKRRKR*GLF (*n* = 2)) in the cleavage site of the HA gene, indicating a high-pathogenicity phenotype in chickens (Supplementary Table S1). The H5N8 clade 2.3.4.4b viruses identified in late October 2020 from a fecal sample of a mandarin duck collected in Chungnam province, South Korea, and a fecal sample of a northern pintail collected in Hokkaido, Japan, also had the PLREKRRKR*GLF cleavage site motif [20,30].

**Figure 2:**
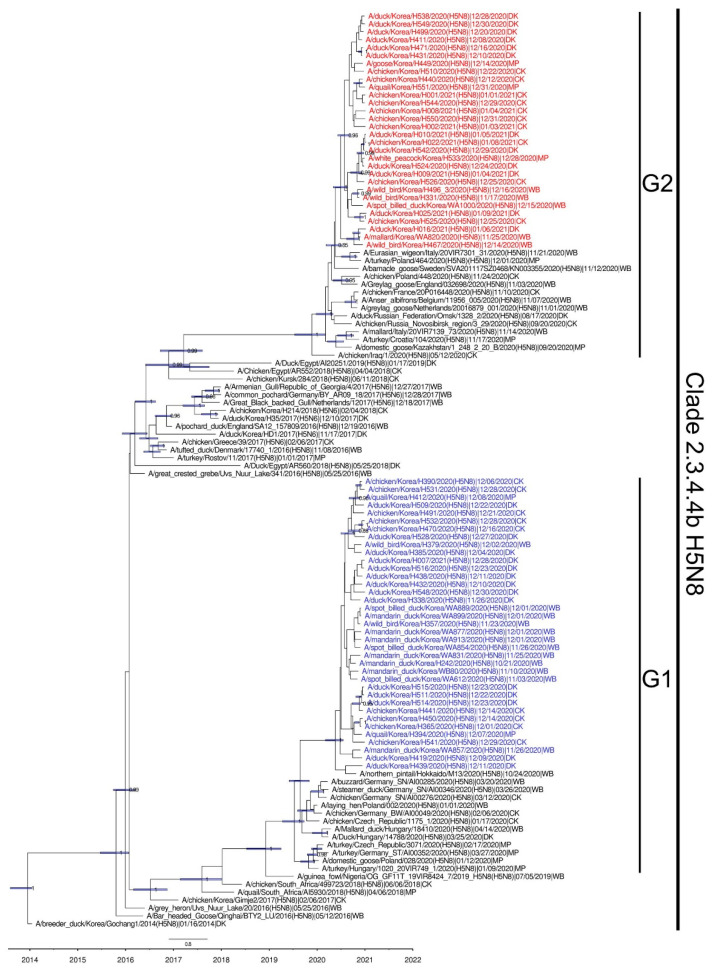
Maximum clade credibility (MCC) trees of hemagglutinin gene of H5N8 highly pathogenic avian influenza viruses isolated in South Korea during the winter of 2020–2021 (time shown on the horizontal axis) were inferred using an uncorrelated lognormal distribution relaxed clock in Beast v1.10.4. We reconstructed the phylogenetic tree with a chain length of 50,000,000 steps, sampling every 5000 steps. For trees with an ESS value >200, MCC trees were generated. Branch lengths represent time. Posterior probabilities >0.8 are provided for key nodes. The G1 and G2 clusters isolated in South Korea are highlighted in blue and red, respectively.

The phylogenetic analysis of the HA gene of the HPAI H5N8 viruses detected in South Korea showed that all the isolates belong to H5 clade 2.3.4.4b and form two distinct genetic groups (G1 and G2), suggesting separate introductions of two genetically distinct H5N8 clade 2.3.4.4 viruses (Figure 2). The initially detected wild bird origin H5N8 clade 2.3.4.4b viruses from South Korea and Japan in late October 2020 belonged to the cluster G1, which was closely related to the H5N8 HPAI virus identified in Poland, Slovakia, and Germany in late 2019–early 2020 [17]. The long branch length between cluster G1 and their closest relatives suggests that the virus had been circulating undetected in wild birds or domestic birds before being introduced into South Korea and Japan. We assume that the H5N8 viruses maintained in wild birds were transmitted from Europe to East Asia via breeding sites through migratory waterfowl. However, the details of the transmission routes of these viruses remain uncertain due to the lack of surveillance data for waterfowl breeding grounds during 2020.

On the other hand, cluster G2 had a close genetic relationship with the H5N8 clade 2.3.4.4b viruses that have been identified in the Republic of Iraq, Italy, and England since May 2020 [19], and shared a common ancestor with the viruses detected in Europe during late 2020, suggesting that it was most likely disseminated through overlapping breeding areas between Europe and South Korea during fall 2020.

**Figure 3:**
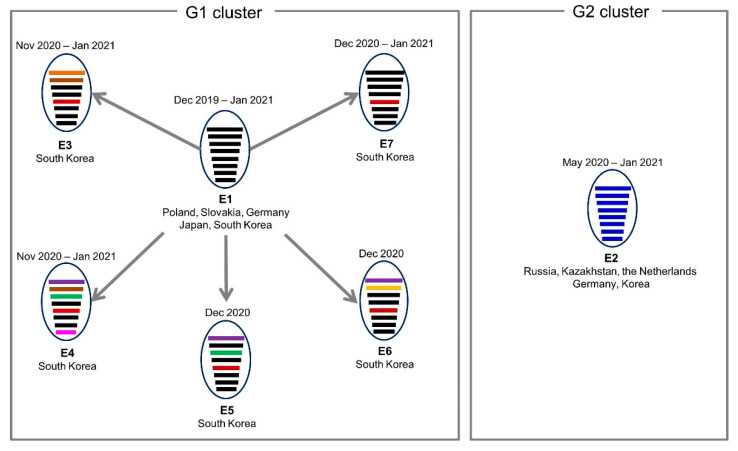
Genome constellation of clade 2.3.4.4b H5N8 highly pathogenic avian influenza (HPAI) viruses in South Korea during the winter of 2020–2021. Oval lines represent the avian virus, and horizontal bars indicate the eight gene segments (from top to bottom): polymerase basic protein 2, polymerase basic protein 1, polymerase acidic protein, hemagglutinin, nucleoprotein, neuraminidase, matrix protein, and non-structural protein. Each color represents a different viral origin: black, late 2019–early 2020 Europe H5N8 HPAI virus [17,18]; blue, late 2020 H5N8 Europe HPAI virus [19]; green, red, purple, orange, dark orange, and cyan blue, Eurasian low pathogenic avian influenza viruses.

The phylogenetic analysis of all the gene segments showed that multiple, distinct genetic subgroups were identified for each gene segment (Supplementary Figure S1). The gene constellation analysis demonstrated that the 67 H5N8 HPAI viruses isolated in South Korea during 2020–2021 were classified into seven distinct genotypes (Figure 3). The consistent clustering of G2 cluster viruses with other H5N8 viruses that circulated in Europe during late 2020 in the phylogenies for each gene (designated as Genotype E2) suggests that the G2 cluster viruses evolved through genetic drift from common ancestor viruses in the absence of further reassortment. By contrast, the G1 cluster viruses showed a high level of heterogeneity in their internal genes. All the genes of the G1 cluster viruses initially found in South Korea in October 2020 clustered together with the H5N8 HPAI viruses detected in Europe during early 2020 (designated as E1). On a genetic backbone of the E1 genotype, the novel reassortants with internal genes of Eurasian LPAIV were designated as E3 to E7 based on the time of detection and genome constellation. Interestingly, all the reassortant genotypes of the G1 cluster (E3–E7) possessed the NP gene segment belonging to the same genetic subgroup. The E1 to E4 genotypes were identified in wild birds (Supplementary Figure S2), and all the seven genotypes (E1–E7) were identified in domestic poultry from approximately 1 month after the initial report of the E1 genotype in wild birds. Although the E5–E7 genotypes were identified in poultry, no poultry origin precursor virus that contributed to the genesis of the reassortant genotypes was identified for these genotypes. In addition, no LPAI virus except the subtype H9N2 was detected in commercial poultry under nationwide surveillance that examined 662,152 samples in poultry farms from 2017 to 2020, suggesting that introductions of these multiple genotypes were likely produced by reassortment in wild waterfowl rather than poultry in Korea. However, the underlying mechanism of the multiple reassortment events in the wild waterfowl population is not fully understood, possibly due to the limited availability of recent data for the wild bird origin influenza viruses in breeding sites. Additional genome sequencing data for H5N8 HPAI viruses isolated from wild birds in Korea would provide insight into the evolutionary history of the viruses, since the present study included only a limited number of H5N8 HPAI virus samples isolated from wild birds.

**Figure 4:**
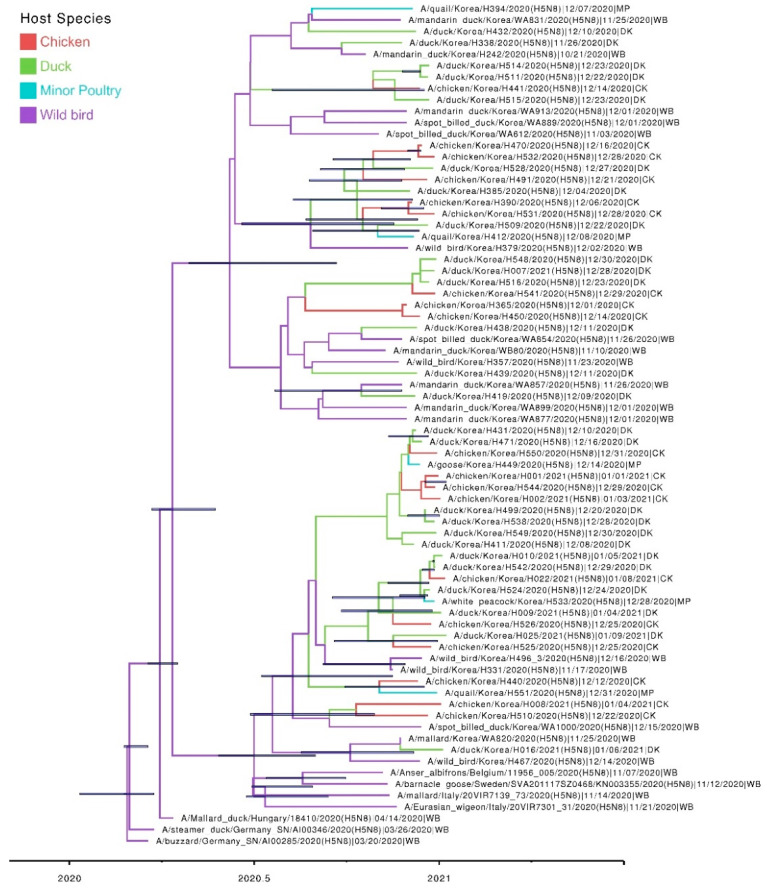
Host transition and MCC tree for HA gene (clusters G1 and G2) of H5N8 highly pathogenic avian influenza virus for 2020–2021 outbreak in South Korea. Coding region of hemagglutinin was used for the reconstruction of the phylogenetic tree. Branches are colored according to host type. Horizontal bars on the nodes indicate the 95% Bayesian credibility intervals of divergence time estimates. Scale bar indicates the branch lengths in a year.

Host transition analysis of the H5N8 clade 2.3.4.4b HPAI viruses during the winter of 2020–2021 was performed by estimating the transition rate, Markov jump count, BF, and posterior probability (Table 1 and Figure 4). Bayesian phylogenetic simulation revealed three major viral transmissions with significant statistical support (a posterior probability >0.5 and a Bayes factor (BF) >4): wild waterfowl to domestic ducks, domestic ducks to chickens, and domestic ducks to minor poultry. Bayesian phylogenetic analysis suggested >13 separate introductions of the H5N8 viruses from wild birds to domestic duck farms (Figure 4). Molecular dating analyses showed that the TMRCA of the G1 and G2 was estimated to be 23 April 2020 (95% Bayesian credible interval: 27 January 2020–9 July 2020), and 14 June 2020 (95% Bayesian credible interval: 4 April 2020–8 August 2020), respectively. The TMRCA of G2 overlapped with the breeding seasons of many wild birds inhabiting Eurasia [31], whereas that of G1 corresponded with both the wintering and breeding seasons. It is likely that the ancestral viruses of G1 and G2 circulated in migratory wild birds from the spring and summer of 2020. After then, the H5N8 viruses spilled over into domestic ducks in South Korea through migratory wild birds during the fall and winter of 2020. In addition, 11 of 17 isolates derived from wild birds were isolated from the healthy captured birds, suggesting that asymptomatically infected wild birds contributed to the spread of H5 HPAI virus. The frequent viral transmissions from wild waterfowl to domestic duck flocks in Korea indicate the vulnerability of domestic duck farms in South Korea to HPAI virus introduction via wild waterfowl rather than domestic chicken farms. Domestic ducks generally show fewer clinical signs upon infection with HPAI viruses, which partly explains why HPAI viruses in domestic ducks may circulate unnoticed and for longer than when infecting chickens [3,32]. Collectively, these results indicate that genetically divergent H5N8 viruses were most likely introduced from wild waterfowl to domestic ducks and then maintained in domestic ducks, followed by the spread of the viruses to domestic chicken and minor poultry farms.

**Figure 5:**
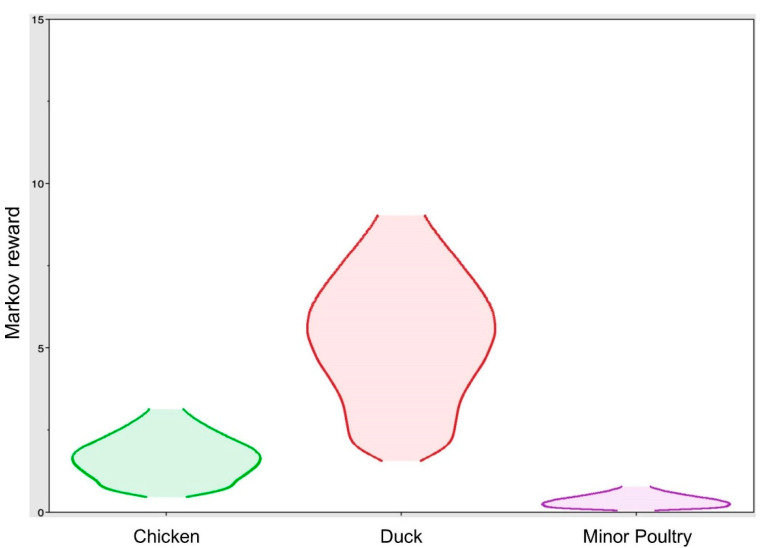
Markov reward time for each poultry species in South Korea. The violin plot of each poultry species (*X*-axis) depicts density distribution of the total time spent in years (*Y*-axis); domestic ducks: 5.53 (95% BCI: 1.62–9.32), chickens: 1.75 (95% BCI: 0.45–3.18), and minor poultry: 0.37 (95% BCI: 0.05–0.79).

The highest mean actual rates and numbers of Markov jumps were observed for transmission from domestic ducks to chickens (transition rate: 2.17; Markov jumps: 13.00), indicating frequent transmission of the viruses from domestic duck farms to chicken farms in South Korea during the outbreak period (Table 1). The estimated total Markov reward time in the poultry types was the highest in domestic ducks (5.53; 95% BCI: 1.62–9.32). The relatively shorter Markov reward time in chickens (1.75, 95% BCI: 0.45–3.18) and minor poultry (0.37; 95% BCI: 0.05–0.79) indicates that H5N8 viruses have been mainly maintained among domestic ducks (Figure 5). Collectively, the frequent viral transmissions from domestic ducks to other poultry types and high Markov reward time suggest that domestic ducks play a major role in the maintenance and spread of viruses among poultry in South Korea. Most duck farms in South Korea have relatively poor biosecurity compared to chicken farms. In particular, because small-holder duck farms belong to sector-three production systems, with low-to-minimal biosecurity, domestic ducks are known to be intermediate hosts between migratory waterfowl and other poultry [33,34]. Consistent with these findings, it has been suggested that, during the 2014–2016 HPAI outbreak in South Korea, H5N8 HPAI initially emerged in western areas (such as Chungnam, Jeonbuk, and Jeonnam Gyeonggi province) of South Korea of high wild bird and domestic duck density and then spread from domestic duck farms to other poultry farms [35,36]. Such data emphasize the need for improved biosafety in domestic duck farms and a decrease in farm density in high-risk areas to prevent further outbreaks of the HPAI virus in South Korea.

Our data suggest that the multiple genotypes containing genomic segments that were genetically closely related to HPAI H5 viruses detected in Europe in 2020 were introduced into South Korea during the fall migration of wild waterfowl and subsequently spilled over into domestic poultry farms. The detection of the H5N8 HPAI viruses in migratory waterfowl and the spread of the viruses via domestic duck farms highlight the importance of the early detection of HPAI viruses in wild waterfowl in wintering sites. In particular, the detection of novel reassortant viruses in domestic duck farms is of great concern because of the potential for long-term maintenance and further spread of the viruses. Thus, surveillance systems for AIV detection in domestic duck farms should be enhanced, especially in the western part of South Korea (a region that is characterized by high wild bird and domestic duck density). Explicit efforts have been made to eradicate HPAI by using a combination of preventive and control measures that include the stamping-out and movement control of poultry in affected areas. Enhanced biosecurity measures at the farm level should be implemented to effectively prevent the introduction of HPAI into poultry farms and the further spread of viruses.

## Figures and Tables

**Table 1:** Transition rates, numbers of Markov jumps, and statistical support values for host types for the HA gene of the H5N8 highly pathogenic avian influenza viruses isolated in South Korea during 2020–2021.

Transition from	Transition to	Mean Actual Migration Rate ^a^ (95% BCI ^b^)	Mean Number of Markov Jumps (95% BCI)	Bayes Factor	Posterior Probability
**Wild bird**	**Duck**	**1.33 (0.12–2.92)**	**9.26 (3–15)**	**10,120.55**	**1.00**
	Chicken	0.04 (0–0.27)	0.13 (0–1)	0.32	0.12
	Minor poultry	0.03 (0–0.17)	0.06 (0–0)	0.25	0.10
**Duck**	Wild bird	0.08 (0–0.57)	0.26 (0–2)	0.52	0.19
	**Chicken**	**2.17 (0.36–4.48)**	**13.00 (9–16)**	**20,243.35**	**1.00**
	**Minor poultry**	**0.81 (0–1.93)**	**4.26 (0–6)**	**44.83**	**0.95**
Chicken	Wild bird	0.06 (0–0.45)	0.06 (0–1)	0.47	0.17
	Duck	0.09 (0–0.59)	0.15 (0–1)	0.53	0.19
	Minor poultry	0.26 (0–1.29)	0.68 (0–4)	1.39	0.38
Minor poultry	Wild bird	0.12 (0–0.65)	0.04 (0–0)	1.31	0.37
	Duck	0.12 (0–0.65)	0.03 (0–0)	1.25	0.36
	Chicken	0.15 (0–0.77)	0.10 (0–1)	1.67	0.43

^a^ Actual migration rates were calculated as the rate x indicator. ^b^ BCI: Bayesian credibility interval. Well-supported viral transitions (a posterior probability >0.5, and a Bayes factor >4) are shown in bold.

## Data Availability

Data is contained within the article, no additional data used.

## Supplementary Materials

The following are available online at https://www.mdpi.com/1999-4915/13/3/490/s1. Supplementary Figure S1. The maximum-likelihood phylogenetic tree for the PB2 (A), PB1 (B), PA (C), NP (D), MP (E), NS (F), and NA (G) genes; Supplementary Figure S2. Chronological distribution of H5N8 viruses according to genotype; Supplementary Table S1. Clade 2.3.4.4b H5N8 highly pathogenic avian influenza viruses isolated in this study. Supplementary Table S2. The virus list from the GISAID Epiflu and GenBank database, on which this research is based.
