# Supplementary material for: Multiple Reassortants of H5N8 Clade 2.3.4.4b Highly Pathogenic Avian Influenza Viruses Detected in South Korea during the Winter of 2020–2021
Source: Viruses. 2021 Mar 16;13(3):490. doi: 10.3390/v13030490 (PMC8001867; doi:10.3390/v13030490)
Supplement: Supplementary file 1 [file viruses-13-00490-s001.zip › viruses-1135196-SI/Supplementary Figure legends.pdf]

Supplementary Figure legend

Supplementary Figure 1. The maximum-likelihood phylogenetic tree for the PB2 (A), PB1 (B), PA (C), NP (D), MP (E), NS (F) and NA (G) genes.

The background color of a label of the viruses in the phylogenetic tree of internal gene and NA gene indicates separate cluster of H5N8 viruses isolated in South Korea during the winter of 2020-2021. The sequences of the PB2, PB1, PA, NP, MP, NS and NA genes comprised 12,251, 12,495, 12,610, 11,153, 10,376, 10,871 and 118 avian influenza viruses since 2000, respectively. Influenza A virus sequences available from the Global Initiative on Sharing All Influenza Data (GISAID) and GeneBank were analyzed using the RAxML phylogeny tool.

Supplementary Figure 2. Chronological distribution of H5N8 viruses according to genotype.

Bars represent the number of H5N8 virus isolates in wild bird (upper) and poultry (lower) according to time and virus genotypes from October 2020 to January 2021 (Dark red. E1; blue, E2; red, E3; orange, E4; pink, E5; purple, E6; black, E7).
