# Supplementary material for: Multiple Reassortants of H5N8 Clade 2.3.4.4b Highly Pathogenic Avian Influenza Viruses Detected in South Korea during the Winter of 2020–2021
Source: Viruses. 2021 Mar 16;13(3):490. doi: 10.3390/v13030490 (PMC8001867; doi:10.3390/v13030490)
Supplement: Supplementary file 1 [file viruses-13-00490-s001.zip › viruses-1135196-SI/Supplementary Table 1.pdf]

Supplementary Table 1. Clade 2.3.4.4b H5N8 highly pathogenic avian influenza viruses isolated in this study

| | Avian species | Virus name | Cleavage site | Collection date | Region | Genetic group | Sample type | latitude | longitude | GISAID PB2 accession | GISAID PB1 accession | GISAID PA accession | GISAID HA accession | GISAID NP accession | GISAID NA accession | GISAID MP accession | GISAID NS accession |
|---|---|---|---|---|---|---|---|---|---|---|---|---|---|---|---|---|---|
| | Duck | A/duck/Korea/H338/2020 | PLREKRRKR*GLF | 2020-11-26 | JB | E1 | Organ | 35°33′52.01″N | 126°46′20.93″E | EPI1845894 | EPI1845895 | EPI1845893 | EPI1845897 | EPI1845890 | EPI1845896 | EPI1845892 | EPI1845891 |
| | Chicken | A/chicken/Korea/H365/2020 | PLREKRRKR*GLF | 2020-12-01 | GB | E1 | Organ | 35°09′19.58″N | 126°36′56.22″E | EPI1845902 | EPI1845903 | EPI1845901 | EPI1845905 | EPI1845898 | EPI1845904 | EPI1845900 | EPI1845899 |
| | Duck | A/duck/Korea/H385/2020 | PLREKRRKR*GLF | 2020-12-04 | JN | E4 | Organ | 34°49′38.3″N | 126°34′38.3″E | EPI1845910 | EPI1845911 | EPI1845909 | EPI1845913 | EPI1845906 | EPI1845912 | EPI1845908 | EPI1845907 |
| | Chicken | A/chicken/Korea/H390/2020 | PLREKRRKR*GLF | 2020-12-06 | GG | E4 | Organ | 37°11′7.85″N | 127°32′33.43″E | EPI1845918 | EPI1845919 | EPI1845917 | EPI1845921 | EPI1845914 | EPI1845920 | EPI1845916 | EPI1845915 |
| | Quail | A/quail/Korea/H394/2020 | PLREKRRKR*GLF | 2020-12-07 | CB | E3 | Organ | 36°59′31.08″N | 127°32′38.97″E | EPI1845926 | EPI1845927 | EPI1845925 | EPI1845929 | EPI1845922 | EPI1845928 | EPI1845924 | EPI1845923 |
| | Duck | A/duck/Korea/H411/2020 | PLREKRRKR*GLF | 2020-12-08 | JN | E2 | Organ | 34°57′27.96″N | 126°43′5.05″E | EPI1845934 | EPI1845935 | EPI1845933 | EPI1845937 | EPI1845930 | EPI1845936 | EPI1845932 | EPI1845931 |
| | Quail | A/quail/Korea/H412/2020 | PLREKRRKR*GLF | 2020-12-08 | GG | E4 | Organ | 37°13′45.25″N | 127°33′39.53″E | EPI1845942 | EPI1845943 | EPI1845941 | EPI1845945 | EPI1845938 | EPI1845944 | EPI1845940 | EPI1845939 |
| | Duck | A/duck/Korea/H419/2020 | PLREKRRKR*GLF | 2020-12-09 | JN | E1 | Organ | 34°53′52.3″N | 126°34′19.21″E | EPI1845950 | EPI1845951 | EPI1845949 | EPI1845953 | EPI1845946 | EPI1845952 | EPI1845948 | EPI1845947 |
| | Duck | A/duck/Korea/H431/2020 | PLREKRRKR*GLF | 2020-12-10 | JN | E2 | Organ | 35°15′29.41″N | 126°41′5.24″E | EPI1845958 | EPI1845959 | EPI1845957 | EPI1845961 | EPI1845954 | EPI1845960 | EPI1845956 | EPI1845955 |
| | Duck | A/duck/Korea/H432/2020 | PLREKRRKR*GLF | 2020-12-10 | JN | E3 | Organ | 35°40′40.02″N | 126°53′53.67″E | EPI1845966 | EPI1845967 | EPI1845965 | EPI1845969 | EPI1845962 | EPI1845968 | EPI1845964 | EPI1845963 |
| | Duck | A/duck/Korea/H438/2020 | PLREKRRKR*GLF | 2020-12-11 | JN | E5 | Organ | 34°49′45.79″N | 126°38′57.99″E | EPI1845974 | EPI1845975 | EPI1845973 | EPI1845977 | EPI1845970 | EPI1845976 | EPI1845972 | EPI1845971 |
| | Duck | A/duck/Korea/H439/2020 | PLREKRRKR*GLF | 2020-12-11 | JN | E6 | Organ | 34°49′43.53″N | 126°38′52.35″E | EPI1845982 | EPI1845983 | EPI1845981 | EPI1845985 | EPI1845978 | EPI1845984 | EPI1845980 | EPI1845979 |
| | Chicken | A/chicken/Korea/H440/2020 | PLREKRRKR*GLF | 2020-12-12 | GG | E2 | Organ | 37°40′21.48″N | 126°35′18.75″E | EPI1845990 | EPI1845991 | EPI1845989 | EPI1845993 | EPI1845986 | EPI1845992 | EPI1845988 | EPI1845987 |
| Poultry | Chicken | A/chicken/Korea/H441/2020 | PLREKRRKR*GLF | 2020-12-14 | JB | E3 | Organ | 35°30′54.94″N | 127°20′23.92″E | EPI1845998 | EPI1845999 | EPI1845997 | EPI1846001 | EPI1845994 | EPI1846000 | EPI1845996 | EPI1845995 |
| | Chicken | A/chicken/Korea/H450/2020 | PLREKRRKR*GLF | 2020-12-14 | GB | E1 | Organ | 36°56′33″N | 127°8′23.08″E | EPI1846006 | EPI1846007 | EPI1846005 | EPI1846009 | EPI1846002 | EPI1846008 | EPI1846004 | EPI1846003 |
| | Goose | A/goose/Korea/H449/2020 | PLREKRRKR*GLF | 2020-12-14 | CN | E2 | Organ | 36°14′17.62″N | 128°16′19.16″E | EPI1846014 | EPI1846015 | EPI1846013 | EPI1846017 | EPI1846010 | EPI1846016 | EPI1846012 | EPI1846011 |
| | Chicken | A/chicken/Korea/H470/2020 | PLREKRRKR*GLF | 2020-12-16 | GG | E4 | Organ | 37°11′6.38″N | 126°47′54.47″E | EPI1846022 | EPI1846023 | EPI1846021 | EPI1846025 | EPI1846018 | EPI1846024 | EPI1846020 | EPI1846019 |
| | Duck | A/duck/Korea/H471/2020 | PLREKRRKR*GLF | 2020-12-16 | JN | E2 | Organ | 35°30′13.16″N | 126°40′50.74″E | EPI1846030 | EPI1846031 | EPI1846029 | EPI1846033 | EPI1846026 | EPI1846032 | EPI1846028 | EPI1846027 |
| | Duck | A/duck/Korea/H499/2020 | PLREKRRKR*GLF | 2020-12-20 | GG | E2 | Organ | 37°9′11.35″N | 127°18′11.05″E | EPI1846038 | EPI1846039 | EPI1846037 | EPI1846041 | EPI1846034 | EPI1846040 | EPI1846036 | EPI1846035 |
| | Chicken | A/chicken/Korea/H491/2020 | PLREKRRKR*GLF | 2020-12-21 | GG | E4 | Organ | 37°10′36.06″N | 127°40′34″E | EPI1846046 | EPI1846047 | EPI1846045 | EPI1846049 | EPI1846042 | EPI1846048 | EPI1846044 | EPI1846043 |
| | Chicken | A/chicken/Korea/H510/2020 | PLREKRRKR*GLF | 2020-12-22 | GG | E2 | Organ | 37°6′39.9″N | 127°41′4.05″E | EPI1846054 | EPI1846055 | EPI1846053 | EPI1846057 | EPI1846050 | EPI1846056 | EPI1846052 | EPI1846051 |
| | Duck | A/duck/Korea/H509/2020 | PLREKRRKR*GLF | 2020-12-22 | CB | E4 | Organ | 37°5′11.02″N | 126°52′51.97″E | EPI1846062 | EPI1846063 | EPI1846061 | EPI1846065 | EPI1846058 | EPI1846064 | EPI1846060 | EPI1846059 |
| | Duck | A/duck/Korea/H511/2020 | PLREKRRKR*GLF | 2020-12-22 | JB | E3 | Organ | 35°29′40.6″N | 127°22′57.02″E | EPI1846070 | EPI1846071 | EPI1846069 | EPI1846073 | EPI1846066 | EPI1846072 | EPI1846068 | EPI1846067 |
| | Duck | A/duck/Korea/H514/2020 | PLREKRRKR*GLF | 2020-12-23 | JB | E3 | Organ | 35°23′41.36″N | 127°20′54.43″E | EPI1846078 | EPI1846079 | EPI1846077 | EPI1846081 | EPI1846074 | EPI1846080 | EPI1846076 | EPI1846075 |
| | Duck | A/duck/Korea/H515/2020 | PLREKRRKR*GLF | 2020-12-23 | JN | E3 | Organ | 35°15′29.29″N | 127°25′49.75″E | EPI1846086 | EPI1846087 | EPI1846085 | EPI1846089 | EPI1846082 | EPI1846088 | EPI1846084 | EPI1846083 |
| | Duck | A/duck/Korea/H516/2020 | PLREKRRKR*GLF | 2020-12-23 | JN | E7 | Organ | 35°15′8.79″N | 127°26′14.72″E | EPI1846094 | EPI1846095 | EPI1846093 | EPI1846097 | EPI1846090 | EPI1846096 | EPI1846092 | EPI1846091 |
| | Duck | A/duck/Korea/H524/2020 | PLREKRRKR*GLF | 2020-12-24 | CN | E2 | Organ | 36°43′49.79″N | 127°14′36.87″E | EPI1846102 | EPI1846103 | EPI1846101 | EPI1846105 | EPI1846098 | EPI1846104 | EPI1846100 | EPI1846099 |

| | | | | | | | | | | | | | | | | | |
|---|---|---|---|---|---|---|---|---|---|---|---|---|---|---|---|---|---|
| | Chicken | A/chicken/Korea/H525/2020 | PLIEKRRKR*GLF | 2020-12-25 | CN | E2 | Organ | 36°43′55.41″N | 126°43′17.02″E | EPI1846110 | EPI1846111 | EPI1846109 | EPI1846113 | EPI1846106 | EPI1846112 | EPI1846108 | EPI1846107 |
| | Chicken | A/chicken/Korea/H526/2020 | PLREKRRKR*GLF | 2020-12-25 | GB | E2 | Organ | 34°54′26.98″N | 129°15′6.87″E | EPI1846118 | EPI1846119 | EPI1846117 | EPI1846121 | EPI1846114 | EPI1846120 | EPI1846116 | EPI1846115 |
| | Duck | A/duck/Korea/H528/2020 | PLREKRRKR*GLF | 2020-12-27 | JB | E4 | Organ | 35°30′42.29″N | 126°47′55.45″E | EPI1846126 | EPI1846127 | EPI1846125 | EPI1846129 | EPI1846122 | EPI1846128 | EPI1846124 | EPI1846123 |
| | Chicken | A/chicken/Korea/H531/2020 | PLREKRRKR*GLF | 2020-12-28 | GG | E4 | Organ | 37°15′31.7″N | 127°33′47.06″E | EPI1846134 | EPI1846135 | EPI1846133 | EPI1846137 | EPI1846130 | EPI1846136 | EPI1846132 | EPI1846131 |
| | Chicken | A/chicken/Korea/H532/2020 | PLREKRRKR*GLF | 2020-12-28 | GG | E4 | Organ | 37°3′28.36″N | 126°53′57.19″E | EPI1846142 | EPI1846143 | EPI1846141 | EPI1846145 | EPI1846138 | EPI1846144 | EPI1846140 | EPI1846139 |
| | Duck | A/duck/Korea/H538/2020 | PLREKRRKR*GLF | 2020-12-28 | JB | E2 | Organ | 37°42′47.34″N | 126°51′2.41″E | EPI1846150 | EPI1846151 | EPI1846149 | EPI1846153 | EPI1846146 | EPI1846152 | EPI1846148 | EPI1846147 |
| | Duck | A/duck/Korea/H007/2020 | PLREKRRKR*GLF | 2020-12-28 | GG | E2 | Organ | 35°56′3.98″N | 126°59′15.26″E | EPI1846447 | EPI1846448 | EPI1846446 | EPI1846450 | EPI1846443 | EPI1846449 | EPI1846445 | EPI1846444 |
| | White peacock | A/white peacoak/Korea/H533/2020 | PLREKRRKR*GLF | 2020-12-29 | CN | E3 | Organ | 36°14′40.16″N | 127°5′3.08″E | EPI1846455 | EPI1846456 | EPI1846454 | EPI1846458 | EPI1846453 | EPI1846451 | EPI1846457 | EPI1846452 |
| | Chicken | A/chicken/Korea/H541/2020 | PLREKRRKR*GLF | 2020-12-29 | GG | E2 | Organ | 35°16′56.74″N | 127°18′53.51″E | EPI1846463 | EPI1846464 | EPI1846462 | EPI1846466 | EPI1846461 | EPI1846459 | EPI1846465 | EPI1846460 |
| | Chicken | A/chicken/Korea/H544/2020 | PLREKRRKR*GLF | 2020-12-29 | JN | E2 | Organ | 37°42′30.41″N | 126°33′17.83″E | EPI1846471 | EPI1846472 | EPI1846470 | EPI1846474 | EPI1846469 | EPI1846467 | EPI1846473 | EPI1846468 |
| | Duck | A/duck/Korea/H542/2020 | PLREKRRKR*GLF | 2020-12-30 | JB | E3 | Organ | 35°25′14.97″N | 126°40′7.1″E | EPI1846479 | EPI1846480 | EPI1846478 | EPI1846482 | EPI1846477 | EPI1846475 | EPI1846481 | EPI1846476 |
| | Duck | A/duck/Korea/H548/2020 | PLREKRRKR*GLF | 2020-12-30 | JB | E2 | Organ | 35°34′45.5″N | 126°42′8.4″E | EPI1846487 | EPI1846488 | EPI1846486 | EPI1846490 | EPI1846485 | EPI1846483 | EPI1846489 | EPI1846484 |
| | Duck | A/duck/Korea/H549/2020 | PLREKRRKR*GLF | 2020-12-31 | GG | E2 | Organ | 37°41′8.56″N | 126°37′33.29″E | EPI1846495 | EPI1846496 | EPI1846494 | EPI1846498 | EPI1846493 | EPI1846491 | EPI1846497 | EPI1846492 |
| | Chicken | A/chicken/Korea/H550/2020 | PLREKRRKR*GLF | 2020-12-31 | GB | E2 | Organ | 35°45′27.1″N | 129°11′47.87″E | EPI1846503 | EPI1846504 | EPI1846502 | EPI1846506 | EPI1846501 | EPI1846499 | EPI1846505 | EPI1846500 |
| | Quail | A/quail/Korea/H551/2020 | PLREKRRKR*GLF | 2021-01-01 | JN | E2 | Organ | 34°54′33.93″N | 126°24′49.48″E | EPI1846511 | EPI1846512 | EPI1846510 | EPI1846514 | EPI1846509 | EPI1846507 | EPI1846513 | EPI1846508 |
| | Chicken | A/chicken/Korea/H001/2021 | PLREKRRKR*GLF | 2021-01-03 | CN | E2 | Organ | 36°57′28.53″N | 127°6′21.24″E | EPI1846519 | EPI1846520 | EPI1846518 | EPI1846522 | EPI1846515 | EPI1846521 | EPI1846517 | EPI1846516 |
| | Chicken | A/chicken/Korea/H002/2021 | PLREKRRKR*GLF | 2021-12-28 | CN | E7 | Organ | 36°49′8.68″N | 127°18′56.43″E | EPI1846527 | EPI1846528 | EPI1846526 | EPI1846530 | EPI1846523 | EPI1846529 | EPI1846525 | EPI1846524 |
| | Chicken | A/chicken/Korea/H008/2021 | PLREKRRKR*GLF | 2021-01-04 | SJ | E2 | Organ | 36°30′48.2″N | 127°22′51.43″E | EPI1846535 | EPI1846536 | EPI1846534 | EPI1846538 | EPI1846531 | EPI1846537 | EPI1846533 | EPI1846532 |
| | Duck | A/duck/Korea/H009/2021 | PLREKRRKR*GLF | 2021-01-04 | JB | E2 | Organ | 35°35′22.83″N | 126°33′28.17″E | EPI1846543 | EPI1846544 | EPI1846542 | EPI1846546 | EPI1846539 | EPI1846545 | EPI1846541 | EPI1846540 |
| | Duck | A/duck/Korea/H010/2021 | PLREKRRKR*GLF | 2021-01-05 | CB | E2 | Organ | 37°3′7″N | 127°31′44.42″E | EPI1846551 | EPI1846552 | EPI1846550 | EPI1846554 | EPI1846547 | EPI1846553 | EPI1846549 | EPI1846548 |
| | Duck | A/duck/Korea/H016/2021 | PLREKRRKR*GLF | 2021-01-06 | JN | E2 | Organ | 34°48′44.24″N | 126°43′9.85″E | EPI1846703 | EPI1846704 | EPI1846702 | EPI1846706 | EPI1846699 | EPI1846705 | EPI1846701 | EPI1846700 |
| | Chicken | A/chicken/Korea/H022/2021 | PLREKRRKR*GLF | 2021-01-08 | GG | E2 | Organ | 37°40′15.73″N | 126°32′58.69″E | EPI1846559 | EPI1846560 | EPI1846558 | EPI1846562 | EPI1846555 | EPI1846561 | EPI1846557 | EPI1846556 |
| | Duck | A/duck/Korea/H025/2021 | PLREKRRKR*GLF | 2021-01-09 | GN | E2 | Organ | 35°42′31.98″N | 128°0′19.32″E | EPI1846567 | EPI1846568 | EPI1846566 | EPI1846570 | EPI1846563 | EPI1846569 | EPI1846565 | EPI1846564 |
| | Mandarin duck | A/mandarin duck/Korea/H242/2020 | PLREKRRKR*GLF | 2020-10-21 | CN | E1 | Feces | 36°45′06.54″N | 127°06′39.72″E | EPI1812356 | EPI1812357 | EPI1812358 | EPI1812359 | EPI1812360 | EPI1812361 | EPI1812362 | EPI1812363 |
| | Spot-billed duck | A/spot-billed duck/Korea/WA612/2020 | PLREKRRKR*GLF | 2020-11-03 | CN | E1 | Swab | 36°45′23.98″N | 127°16′01.74″E | EPI1846575 | EPI1846576 | EPI1846574 | EPI1846578 | EPI1846573 | EPI1846571 | EPI1846577 | EPI1846572 |
| Wild bird | Mandarin duck | A/mandarin duck/Korea/WB80/2020 | PLREKRRKR*GLF | 2020-11-10 | GG | E1 | Swab | 37°13′52″N | 127°26′20″E | EPI1846583 | EPI1846584 | EPI1846582 | EPI1846586 | EPI1846581 | EPI1846579 | EPI1846585 | EPI1846580 |
| | Wild duck | A/wild duck/Korea/H331/2020 | PLREKRRKR*GLF | 2020-11-17 | JJ | E2 | Feces | 33°30′20.93″N | 126°53′30.47″E | EPI1846695 | EPI1846696 | EPI1846694 | EPI1846698 | EPI1846693 | EPI1846691 | EPI1846697 | EPI1846692 |
| | Wild duck | A/wild duck/Korea/H357/2020 | PLREKRRKR*GLF | 2020-11-23 | JB | E3 | Feces | 35°44′0″N | 126°40′36″E | EPI1846591 | EPI1846592 | EPI1846590 | EPI1846594 | EPI1846589 | EPI1846587 | EPI1846593 | EPI1846588 |
| | Mallard | A/mallard/Korea/WA820/2020 | PLREKRRKR*GLF | 2020-11-25 | GG | E2 | Swab | 37°06.′59.88″N | 127°24′21.33″E | EPI1846599 | EPI1846600 | EPI1846598 | EPI1846602 | EPI1846597 | EPI1846595 | EPI1846601 | EPI1846596 |

| | | | | | | | | | | | | | | | | |
|---|---|---|---|---|---|---|---|---|---|---|---|---|---|---|---|---|
| Mandarin duck | A/mandarin duck/Korea/WA831/2020 | PLREKRRKR*GLF | 2020-11-25 | JB | E3 | Swab | 35°41′03.23″N | 126°51′44.94″E | EPI1846607 | EPI1846608 | EPI1846606 | EPI1846610 | EPI1846605 | EPI1846603 | EPI1846609 | EPI1846604 |
| Spot-billed duck | A/spot-billed duck/Korea/WA854/2020 | PLREKRRKR*GLF | 2020-11-26 | GG | E3 | Swab | 36°58′41.24″N, | 127°11′23.75″E | EPI1846615 | EPI1846616 | EPI1846614 | EPI1846618 | EPI1846613 | EPI1846611 | EPI1846617 | EPI1846612 |
| Mandarin duck | A/mandarin duck/Korea/WA857/2020 | PLREKRRKR*GLF | 2020-11-26 | JB | E3 | Swab | 35°41′03.23″N | 126°51′44.94″E | EPI1846623 | EPI1846624 | EPI1846622 | EPI1846626 | EPI1846621 | EPI1846619 | EPI1846625 | EPI1846620 |
| Mandarin duck | A/mandarin duck/Korea/WA877/2020 | PLIEKRRKR*GLF | 2020-12-01 | JN | E1 | Swab | 37°11′38.60″N | 127°00′45.41″E | EPI1846631 | EPI1846632 | EPI1846630 | EPI1846634 | EPI1846629 | EPI1846627 | EPI1846633 | EPI1846628 |
| Spot-billed duck | A/spot-billed duck/Korea/WA889/2020 | PLREKRRKR*GLF | 2020-12-01 | GG | E3 | Swab | 36°17′35.77″N | 127°08′44.68″E | EPI1846639 | EPI1846640 | EPI1846638 | EPI1846642 | EPI1846637 | EPI1846635 | EPI1846641 | EPI1846636 |
| Mandarin duck | A/mandarin duck/Korea/WA899/2020 | PLREKRRKR*GLF | 2020-12-01 | CN | E3 | Swab | 36°17′28.85″N | 127°08′46.52″E | EPI1846647 | EPI1846648 | EPI1846646 | EPI1846650 | EPI1846645 | EPI1846643 | EPI1846649 | EPI1846644 |
| Mandarin duck | A/mandarin duck/Korea/WA913/2020 | PLREKRRKR*GLF | 2020-12-01 | CN | E1 | Swab | 36°17′32.46″N | 128°8′6.18″E | EPI1846655 | EPI1846656 | EPI1846654 | EPI1846658 | EPI1846653 | EPI1846651 | EPI1846657 | EPI1846652 |
| Wild duck | A/wild duck/Korea/H379/2020 | PLREKRRKR*GLF | 2020-12-02 | GB | E4 | Feces | 35°55′51.1134″N | 129°14′22.89″E | EPI1846663 | EPI1846664 | EPI1846662 | EPI1846661 | EPI1846666 | EPI1846659 | EPI1846665 | EPI1846660 |
| Wild duck | A/wild duck/Korea/H467/2020 | PLREKRRKR*GLF | 2020-12-14 | CB | E2 | Feces | 36°40′19.5996″N | 127°23′38.82″E | EPI1846671 | EPI1846672 | EPI1846670 | EPI1846674 | EPI1846669 | EPI1846667 | EPI1846673 | EPI1846668 |
| Spot-billed duck | A/spot-billed duck/Korea/WA1000/2020 | PLREKRRKR*GLF | 2020-12-15 | GG | E2 | Swab | 37°13′13.94″N | 127°01′14.97″E | EPI1846679 | EPI1846680 | EPI1846678 | EPI1846682 | EPI1846677 | EPI1846675 | EPI1846681 | EPI1846676 |
| Wild duck | A/wild duck/Korea/H496-3/2020 | PLREKRRKR*GLF | 2020-12-16 | JJ | E2 | Feces | 33°30′20.72″N | 126°53′31.98″E | EPI1846687 | EPI1846688 | EPI1846686 | EPI1846690 | EPI1846685 | EPI1846683 | EPI1846689 | EPI1846684 |

DK, duck; CK, chicken; MP, minor poultry; WB, wild duck; JB, Jeonbuk; GB, Gyeongbuk; JN, Jeonnam; GG, Gyeonggi; CB, Chungbuk; CN, Chungnam; SJ, Sejong; JJ, Jeju;
All of the captured wild birds were healthy status
Wild duck in “species” coulumn means mallard or spot-billed duck in DNA barcoding system
