# Supplementary figures and images for: Multiple Reassortants of H5N8 Clade 2.3.4.4b Highly Pathogenic Avian Influenza Viruses Detected in South Korea during the Winter of 2020–2021
Source: Viruses. 2021 Mar 16;13(3):490. doi: 10.3390/v13030490 (PMC8001867; doi:10.3390/v13030490)

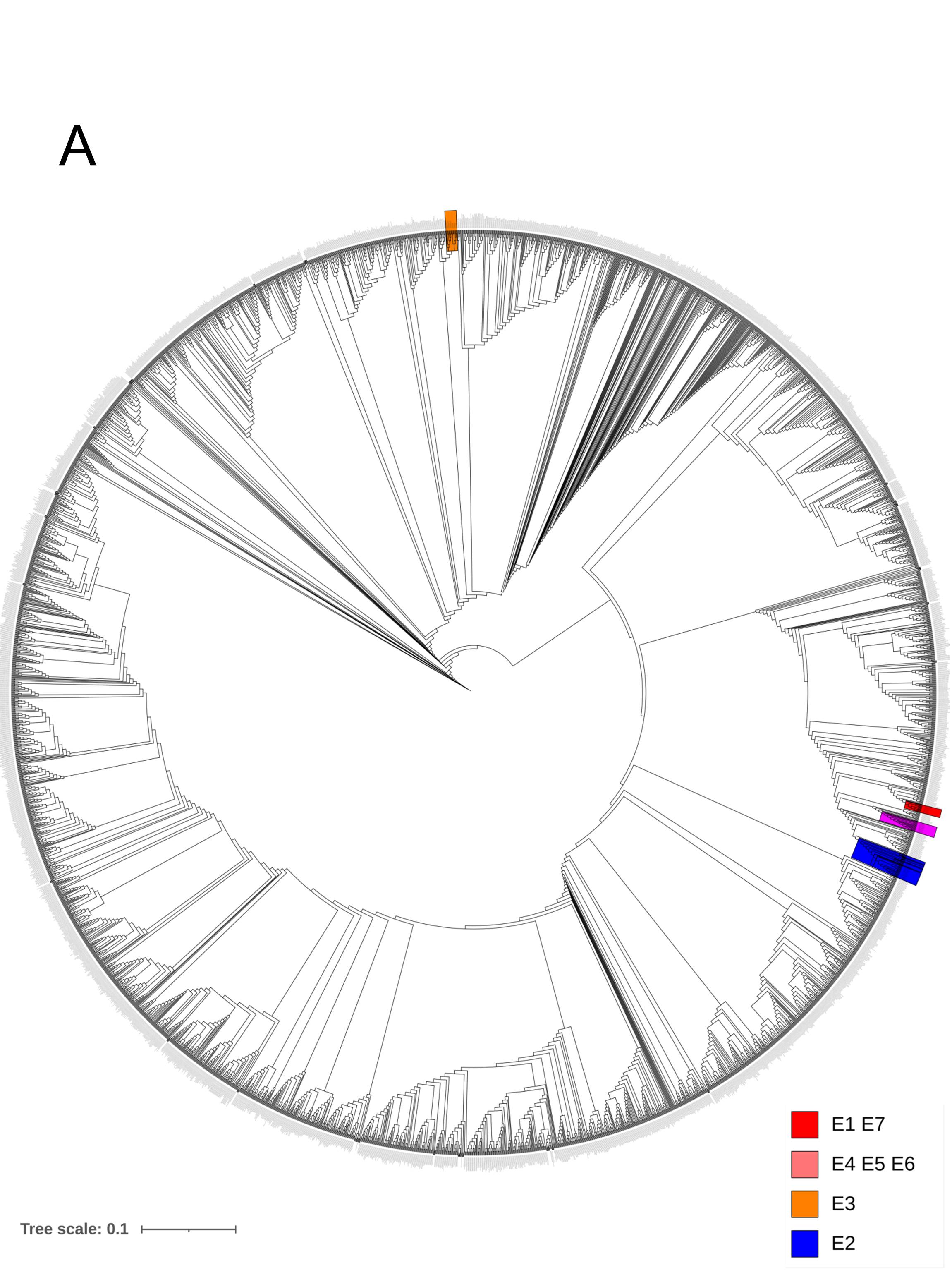

Supplement: Supplementary file 1 [file viruses-13-00490-s001.zip › viruses-1135196-SI/Supplementary Figure 1A.JPG]

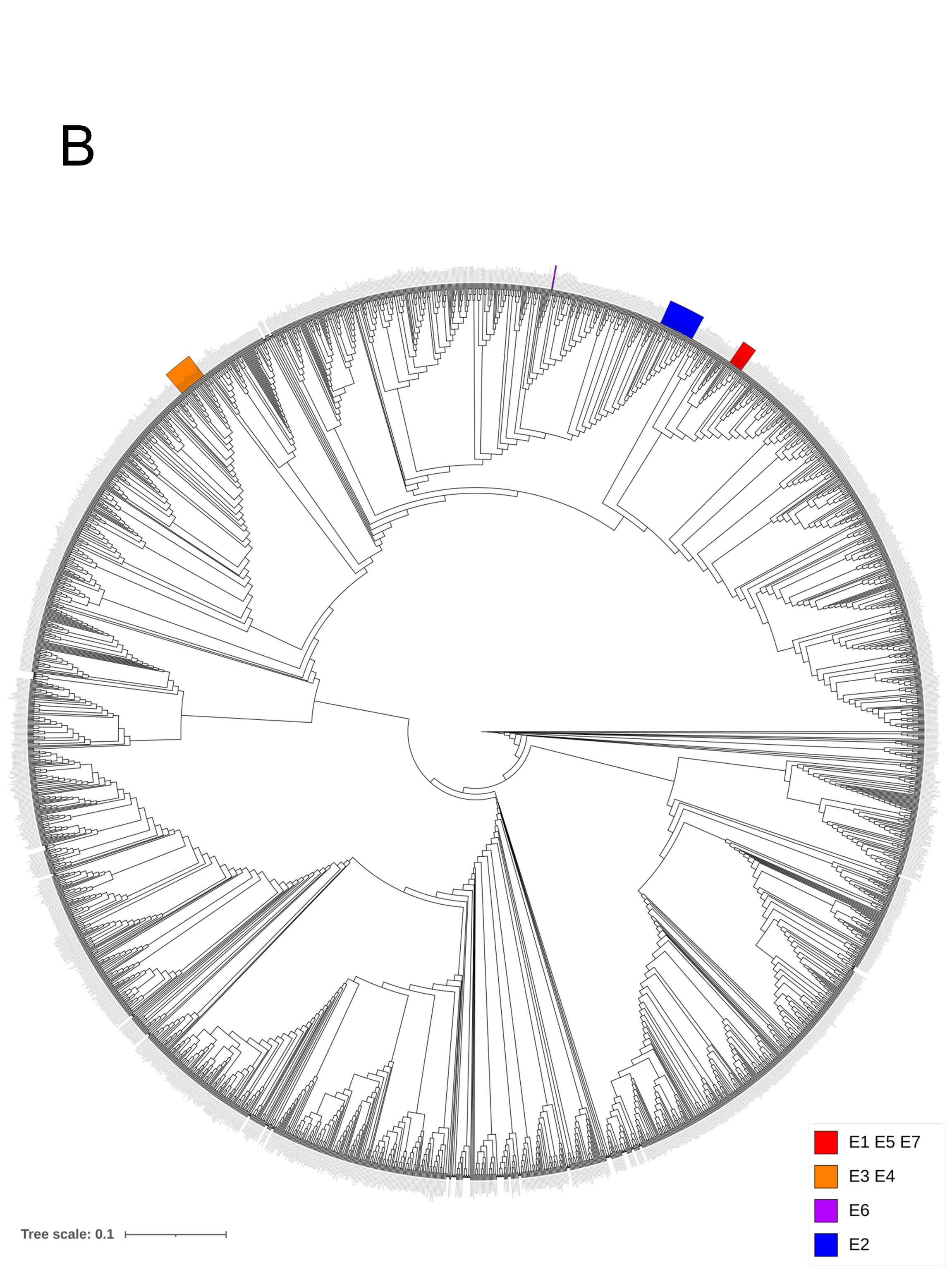

Supplement: Supplementary file 1 [file viruses-13-00490-s001.zip › viruses-1135196-SI/Supplementary Figure 1B.JPG]

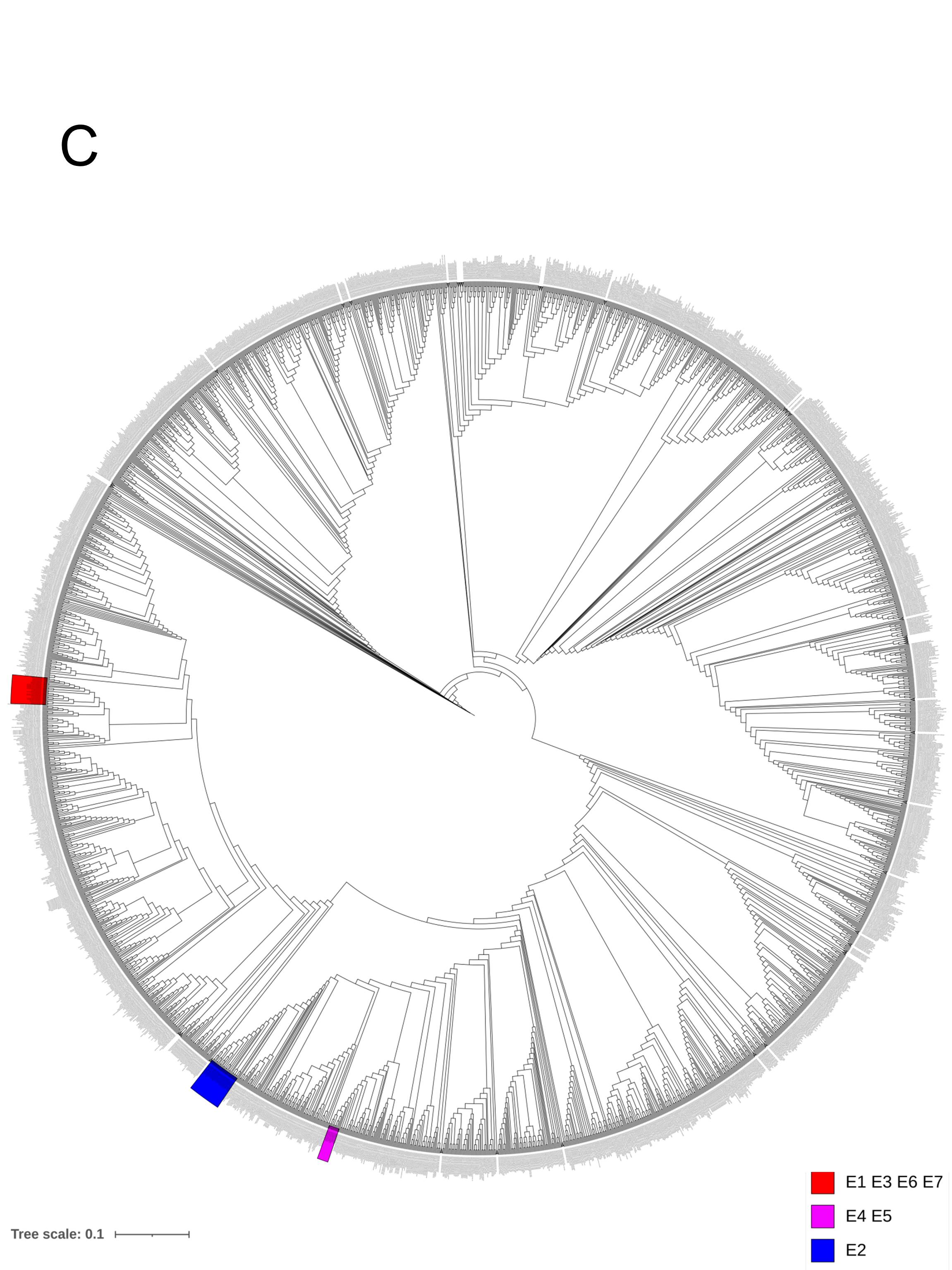

Supplement: Supplementary file 1 [file viruses-13-00490-s001.zip › viruses-1135196-SI/Supplementary Figure 1C.JPG]

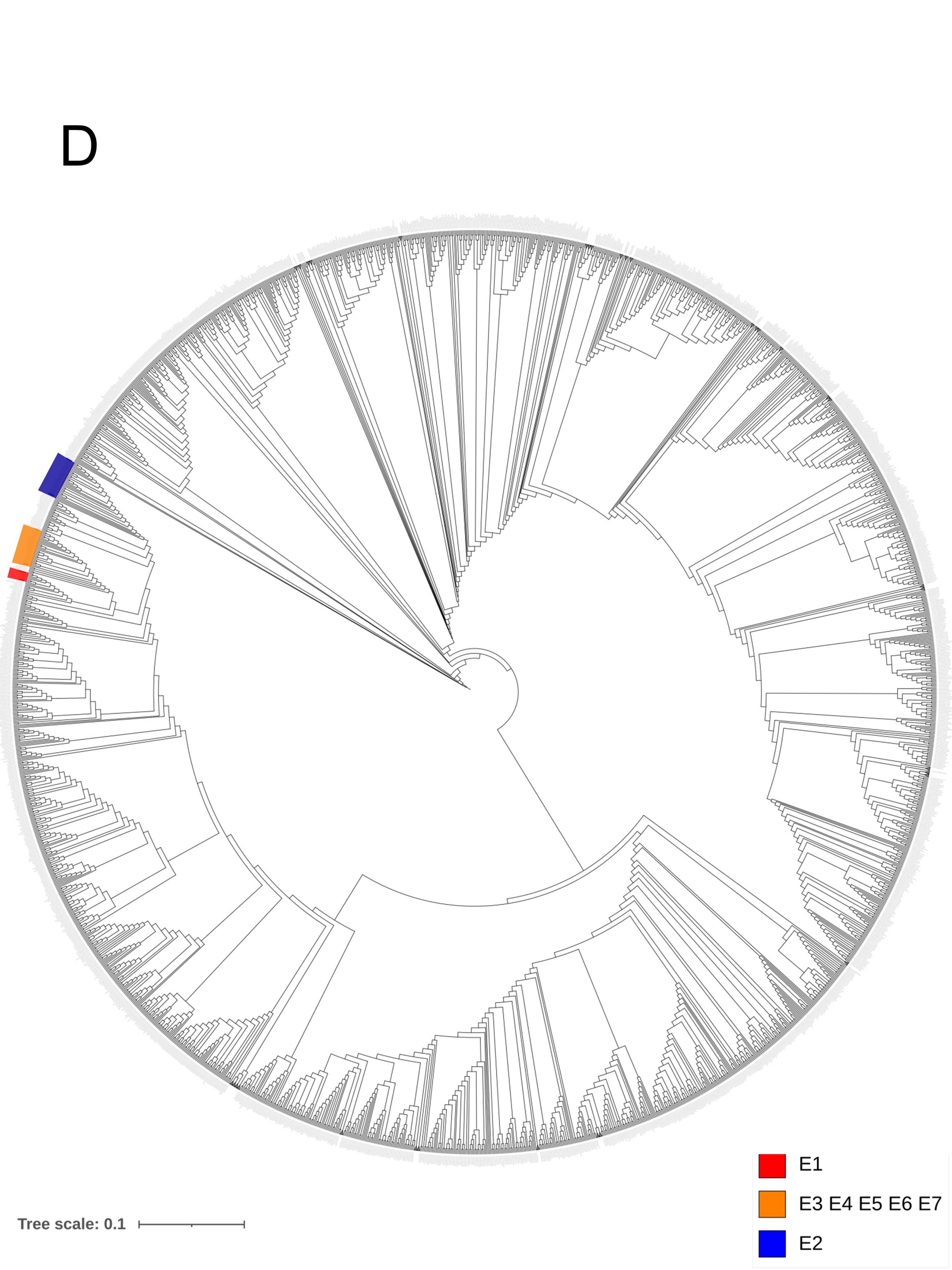

Supplement: Supplementary file 1 [file viruses-13-00490-s001.zip › viruses-1135196-SI/Supplementary Figure 1D.JPG]

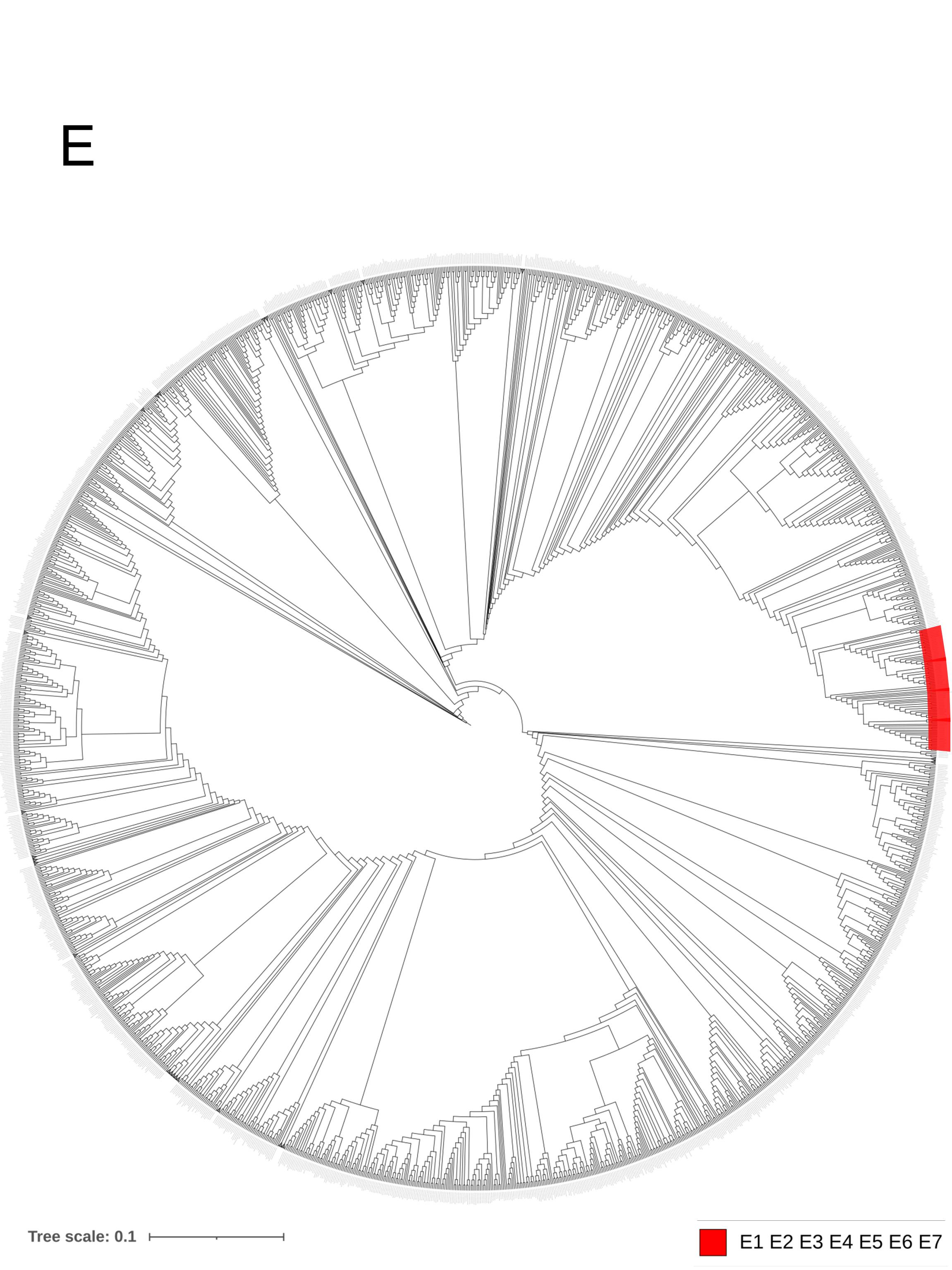

Supplement: Supplementary file 1 [file viruses-13-00490-s001.zip › viruses-1135196-SI/Supplementary Figure 1E.JPG]

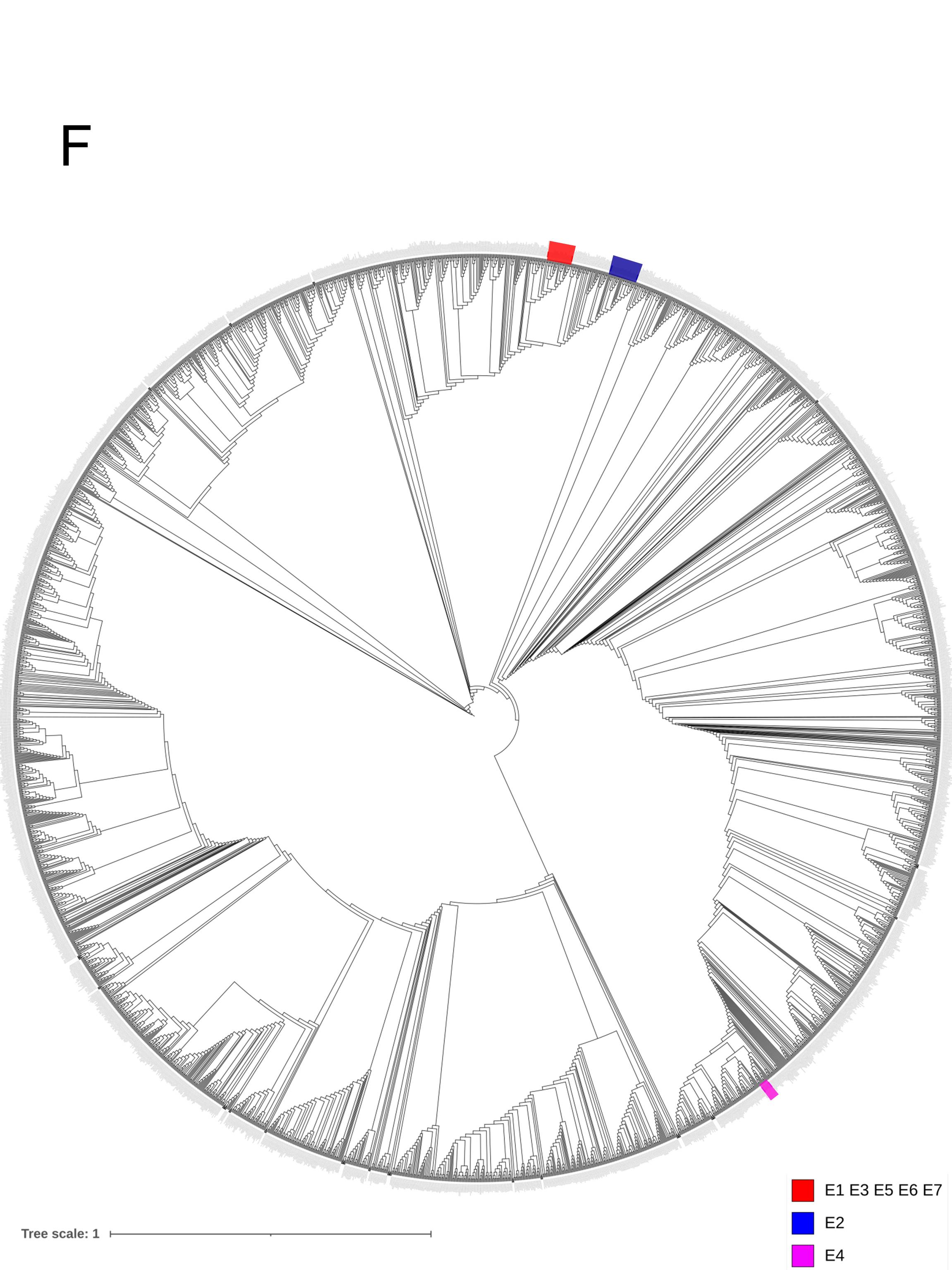

Supplement: Supplementary file 1 [file viruses-13-00490-s001.zip › viruses-1135196-SI/Supplementary Figure 1F.JPG]

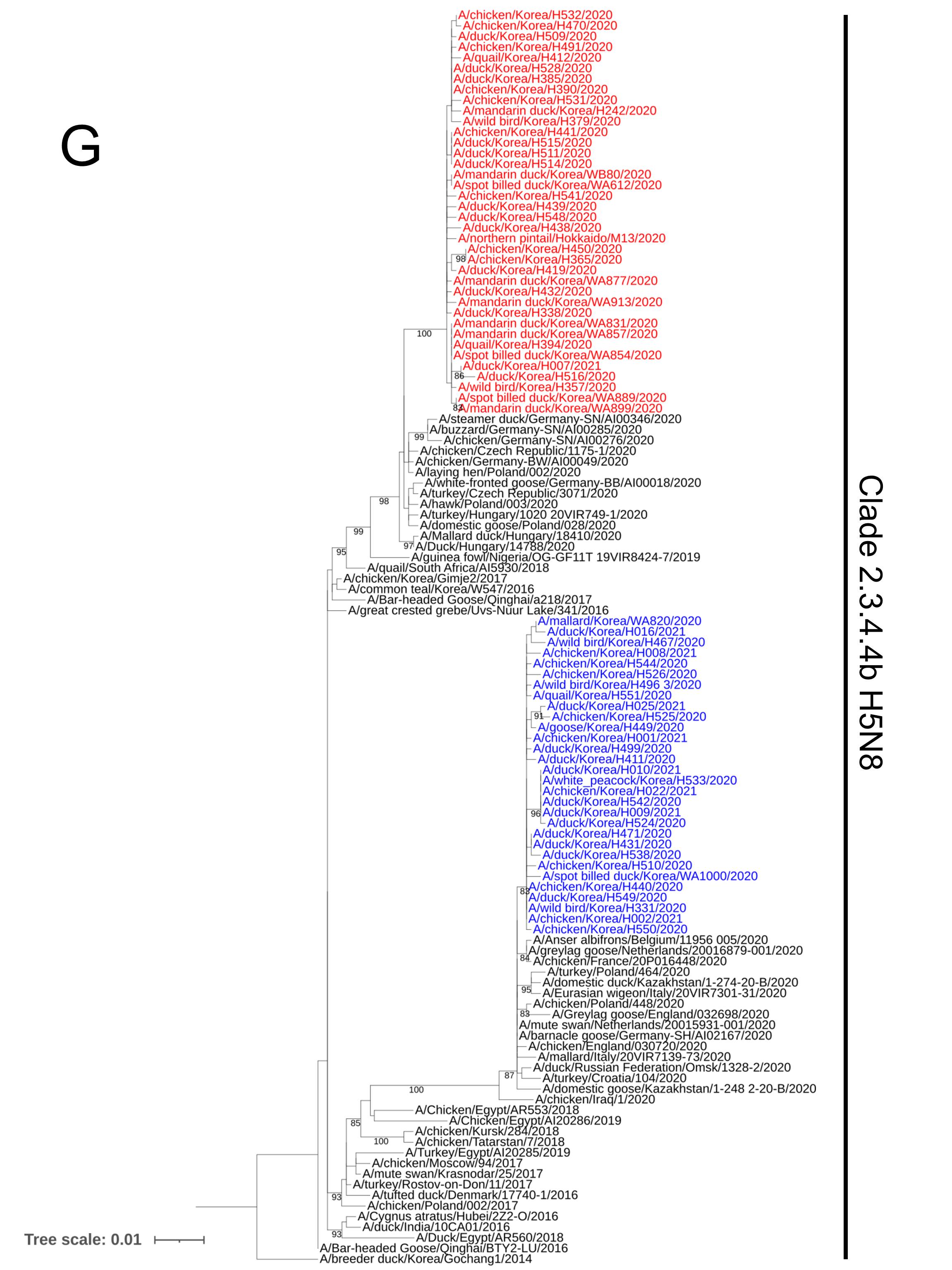

Supplement: Supplementary file 1 [file viruses-13-00490-s001.zip › viruses-1135196-SI/Supplementary Figure 1G.JPG]

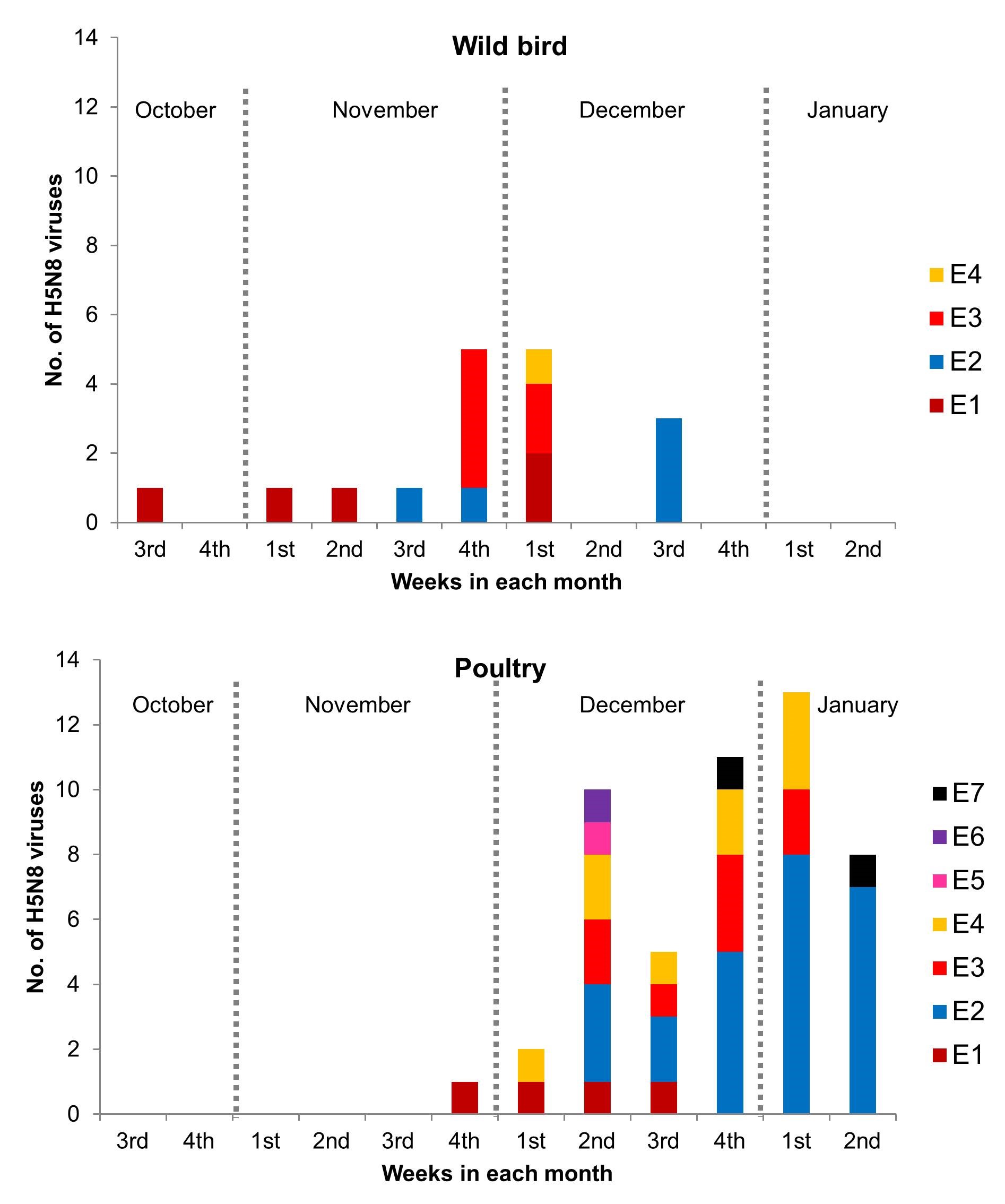

Supplement: Supplementary file 1 [file viruses-13-00490-s001.zip › viruses-1135196-SI/Supplementary Figure 2.jpg]
